# A randomized, blinded, controlled USA field study to assess the use of fluralaner topical solution in controlling feline flea infestations

**DOI:** 10.1186/s13071-017-1972-4

**Published:** 2017-01-19

**Authors:** Cheyney Meadows, Frank Guerino, Fangshi Sun

**Affiliations:** Merck Animal Health, Madison, NJ USA

**Keywords:** Bravecto, Fluralaner, Fleas, Fipronil-methoprene, Cats

## Abstract

**Background:**

Fleas are a common ectoparasite of domestic cats and there is a need for novel treatments that improve feline flea control.

**Methods:**

This investigator-blinded, multi-center randomized, positive-controlled study evaluated the flea control in cats provided by a single owner-applied treatment with a fluralaner topical formulation compared with a positive control. Households with up to five healthy cats, all at least 12 weeks of age and weighing at least 1.2 kg (2.6 lb), were randomized in an approximate 3:1 ratio of fluralaner to positive control. All cats in households randomized to the positive control group were dispensed three treatments, at 4-week intervals, of a commercial formulation of fipronil/(*S*)-methoprene. All cats in households randomized to the fluralaner group were dispensed an initial treatment at enrollment and a second treatment at week 12 for an additional 3-week observation of treatment safety. One primary cat with at least five live fleas at enrollment was randomly selected within each household. Flea counts were performed on all primary cats at 4-week intervals through week 12. Efficacy measurement was based on reduction in flea counts from baseline. Treatment was considered effective at weeks 4, 8 and 12 if mean live flea count reductions were 90% or greater and statistically significantly different (*P* ≤ 0.05) from counts at enrollment.

**Results:**

In 18 investigational veterinary clinics across 11 USA states, 116 households (224 cats) were randomized to receive topical fluralaner and 45 households (87 cats) were randomized to the fipronil-methoprene combination. Fluralaner was demonstrated to be effective at 4 weeks (99.1% flea count reduction), 8 weeks (99.5%), and 12 weeks (99.0%), and all reductions were significantly different from the enrollment count (all *P* < 0.0001). The fipronil-methoprene combination was < 90% effective at each post-treatment assessment, with peak efficacy of 75.4% at 12 weeks (all *P* < 0.0001). No treatment-related serious adverse events were reported in either group.

**Conclusions:**

Owner-applied fluralaner topical treatment was safe in cats and was highly effective in killing fleas over the subsequent 12 weeks.

## Background

Infestation of cats with the flea *Ctenocephalides felis* can be a cause of irritation, leading to pruritus, erythema, excoriations arising from self-mutilation through scratching, and development of papules to produce the condition of feline miliary dermatitis [1]. The rapid accumulation of large-scale flea challenge in any household can be attributed to several factors: the ability of an adult female flea to produce as many as 50 eggs per day; the potentially short flea life-cycle which can be completed in as little as 12 to 14 days; and optimal conditions of humidity and temperature that favor a short flea prepatent period [2]. Thus, absence of adherence to an effective flea control program in cats can quickly lead to a household flea problem.

Fleas have been incriminated as the main vector of cat to human transmission of *Bartonella* species*,* including *Bartonella felis*, the causative agent of cat scratch disease [3]. Fleas have also been shown to be carriers of other potentially zoonotic organisms, including *Rickettsia felis* and *Rickettsia typhi*, and the cestode *Dipylidium caninum*. This tapeworm requires rigorous flea treatment for control, rather than reliance solely on direct cestodicidal chemotherapy [3–5]. Effective flea control is therefore important to reduce the challenge this parasite presents to household pets and their human cohabitants.

The isoxazolines represent a novel family of antiparasitics that provide safe and effective tick and flea protection for dogs. Fluralaner (Bravecto®; Merck Animal Health) is the only isoxazoline that provides 12 weeks of immediate and persistent flea and tick efficacy for dogs [6–9]. Field studies in the USA, Europe and Australia demonstrated the safety and efficacy of orally administered fluralaner for reducing flea and tick infestations on dogs [10–12]. A recent study showed that under field conditions, a topical fluralaner formulation provides a level of flea control in dogs that aligns with that provided by the oral product [13].

To date, no isoxazoline has been shown to be safe and effective for cats. Fluralaner topical solution has favorable pharmacokinetics in cats and can provide an easily administered option for flea and tick control on cats [14]. A field study was therefore designed to assess the flea control efficacy of topical fluralaner dispensed to cat owners for at-home administration in the USA.

The primary objective of this positive-controlled, randomized, single-blinded study was to investigate the clinical safety and efficacy of topical fluralaner to treat and control natural flea infestations for at least 12 weeks (84 days). The results were compared to a group treated with a positive control at 4-week intervals and followed for 12 weeks (84 days) using a commercial formulation of fipronil/(*S*)-methoprene (Frontline® Plus for Cats; Merial). Following the 12 week efficacy evaluation, cats treated with fipronil-methoprene combination were off the study; cats assigned to topical fluralaner were administered a second dose of fluralaner and followed for 3 more weeks (through Day 105, week 15) for safety observations.

## Methods

The study protocol, finalized in early 2013, used then-current guidelines for evaluating flea and tick parasiticides [15]. It also complied with Good Clinical Practice (VICH GL9) and the International Guiding Principles for Biomedical Research Involving Animals. Written informed consent was obtained from each owner for all household cats prior to the commencement of any screening activities. Enrollment eligibility included households that had no more than five cats, all of which were at least 12 weeks of age, weighed at least 1.2 kg (2.6 lb), and were in generally good health as determined by a veterinary examination prior to enrollment; and at least one cat in the household had a minimum of five live fleas counted prior to enrollment. There were no breed or gender restrictions, but households with pregnant or lactating cats were not eligible for enrollment. Households in which the cats had exposure to non-confined pets, other than cats, that could harbor fleas (e.g. dogs) were not eligible. There were also restrictions on the pre-enrollment/historical use of any approved (in the USA, as this was a USA study) flea control medications or products, based on the approved label duration. Products labeled for 12-week use had a minimum 84-day washout, products labeled for monthly use had a minimum 30-day washout, products labeled for use every 2 weeks had a minimum 14-day washout, and products labeled for weekly use had a 7-day washout.

At each of the 18 participating investigational veterinary clinics, enrolled households were randomly assigned between two treatment groups in a 3:1 (fluralaner topical:fipronil-methoprene combination) ratio. No single clinic was permitted to contribute more than 40% of the households that participated in the study in either treatment group. The treatment groups were:

1. Fluralaner topical solution for cats was dispensed for owner administration on Day 0. The efficacy assessment phase of the study lasted for 12 weeks. A second fluralaner topical solution dose was dispensed 12 weeks after the first for additional safety assessment. At least 100 households were targeted for assignment to the topical fluralaner group. Topical fluralaner was provided as a 28% *w/v* solution in single-dose applicators in three sizes, containing either 112.5 mg, 250 mg or 500 mg of fluralaner, in volumes of 0.4, 0.89 and 1.79, respectively, with dosing weight bands established to achieve a minimum dose of 40 mg/kg.
2. Fipronil/(*S*)-methoprene spot-on solution (Frontline® Plus for Cats; Merial Limited, Duluth, GA), was dispensed for application once every 28 days for three doses. A minimum of 33 households were targeted for enrollment into this group. The fipronil-methoprene combination (containing 9.8% fipronil and 11.8% (*S*)-methoprene) remained in its commercial packaging containing a volume of 0.5 ml.

At each site, households were assigned to treatment according to a randomized complete block design, with order of entry into the study as the blocking factor and assignment of cats to treatment within blocks in a ratio of 3:1 of fluralaner-treated to fipronil-methoprene-treated. A primary cat from each household was randomly selected from cats with at least five live fleas. Separate randomization tables were provided to each site for assignment of households to treatment group and selection of the primary cat. All cats within an enrolled household were assigned to the same treatment group.

Each clinic had at least one dispensing administrator who had access to randomization tables and was responsible for dispensing all treatments to owners. Administrators did not participate in the collection or recording of flea count data or the assessment of flea allergy dermatitis (FAD). Study personnel who participated in the collection or recording of flea count data or the assessment of FAD through the final visit were blinded to treatment assignment.

All treatments were administered at home by cat owners, who were not blinded. For households randomized to the fluralaner group, owners were provided instructions on application, including parting the hair and placing the tip on the skin at the base of the skull and squeezing out the contents onto one or more spots in amounts that would limit risk of run-off from any one spot. After administration, the owner was instructed to observe the cat to determine if any of the solution ran or dripped off the animal during or immediately following treatment. Owners of cats randomized to the fipronil-methoprene group were instructed to dose according to label directions.

Enrollments were completed during the first clinic visit, during which treatments were dispensed and blood and urine samples collected for baseline clinical pathology data. Owners were required to bring their cats into the clinic for recheck visits approximately 4, 8, and 12 weeks after enrollment (on Days 28 [± 2], 56 [± 3], and 84 [± 3]). Blood and urine samples were collected for clinical pathology data for all cats at week 12. Households assigned to fipronil-methoprene were off study after the week 12 visit. For households randomized to the fluralaner group, cats were also rechecked for assessments of safety (including a 3^rd^ blood and urine sample collection for clinical pathology) and signs of FAD on week 15 (Day 105 [± 3], 3 weeks after the second fluralaner treatment).

From enrollment until week 12 (Day 84) of the study, owners were asked to avoid any premises treatments for environmental flea control, either in their house or on their property. No concomitant treatments for flea and/or tick infestations were permitted, and the investigator or designee was asked to observe an adequate washout period (consistent with any USA-approved labeling) for any such treatments received prior to enrollment. Concomitant treatments for disorders other than flea and tick infestations were permitted, if they were not expected to interfere with assessments of flea and tick infestations. For example, treatments for prevention and control of internal parasite infestations (including heartworm and gastrointestinal parasites) were permitted, if any product used was not labeled for flea or tick control. Treatment that could affect assessment of signs of FAD (for example steroids, antihistamines, creams, ointments, baths, etc.) was permissible. However, data from any such treated cat were subsequently excluded from the analysis of FAD clinical signs. Grooming, bathing, swimming, and other water activities were permitted during the study, with some exceptions. To avoid any effect on recovery of fleas and ticks, grooming and bathing were disallowed within 72 h before any scheduled recheck visit through week 12 (Day 84). In addition, bathing, swimming, and other water activities were not allowed for 72 h after application of any study treatment.

Owners were instructed to observe their cats for any adverse events and to document such observations in a study-provided diary and to report them as soon as they occurred or at the next scheduled visit. Flea counting was conducted in clinics by blinded personnel, using a flea comb to perform a full-body flea count for at least 10 min; live fleas were counted, removed, and placed in a soap solution. After this 10 min period the flea counter was permitted to stop when they were confident that all fleas had been recovered.

Examinations at each visit included a veterinarian’s assessment of the presence of six signs of FAD (erythema, alopecia, papules, scales, crusts, and excoriations) and their severity (mild, moderate, severe). Pruritus observations were recorded by owners and reported.

The primary efficacy endpoint was based on reduction in mean flea counts compared to baseline, with household as the experimental unit, represented by one primary cat.

### Efficacy assessment

Arithmetic and geometric mean live flea counts were calculated separately for each treatment group at each assessment and the percentage reduction at each time point was based on a comparison to baseline according to the formula:

$$ \mathrm{Percent}\kern0.5em \mathrm{efficacy}=\left(1-\frac{{\mathrm{D}}_{\mathrm{x}}}{{\mathrm{D}}_0}\right)\times 100 $$

where D_0_ = mean live flea count at baseline of primary cats; and D_x_ = mean live flea count on Day x (x = 28 [4 weeks], 56 [8 weeks] or 84 [12 weeks]) of primary cats.

Both geometric means and arithmetic means were compared using the live flea counts and log-transformed live flea counts (log[live flea count + 1]) for each household (represented by one primary cat). A mixed linear model with repeated measures was used for the analysis. The model included treatment, visit and treatment*visit as fixed effects, site as a random effect and household as the subject with repeated measures. A Kenward-Rogers adjustment was used to determine the denominator degrees of freedom for hypothesis testing. Akaike’s Information Criterion (AIC) was used as the criterion to select the covariance structure for repeated measures. Least squares means were used for comparisons and for the log-transformed data, the least squares means were back transformed to obtain the estimates of geometric mean live flea counts. Within each treatment group, the live flea counts at each post-treatment visit (Day 28, 56 and 84) were compared with that at the baseline (Visit 1). At each visit, live flea counts were compared between the two treatment groups. Two-sided t-tests at a 5% level of significance were used for all pairwise comparisons. SAS version 9.3 was the primary software used for analysis. Treatment was considered effective at a given time point if the mean (geometric or arithmetic) live flea count reduction was 90% or greater and significantly different (*P* ≤ 0.05) from baseline [15].

Numbers and percentages of primary cats that showed at least 90% reduction in flea burden, as well as the numbers and percentages of primary cats with zero fleas counted in each treatment group at each visit were calculated. A non-parametric asymptotic approach was used to test the differences of the percentages between treatment groups. The non-parametric analyses were performed using StatXact version 9. The study was only designed to perform statistical comparisons of the flea counts. Signs of FAD and AEs were only examined descriptively. Thus, no *P*-values were presented for these outcomes.

## Results

Between May and December 2013, 311 cats from 161 households were enrolled at 18 sites across 11 states: Alabama (one site), Florida (two sites), Illinois (two sites), Maine (one site), Louisiana (two sites), Missouri (one site), New Jersey (one site), Pennsylvania (three sites), Rhode Island (one site), Tennessee (one site), and Texas (three sites). There were 116 households (i.e. 116 primary cats) with a total of 224 cats randomized to the topical fluralaner group (43.1% were single-cat households), and 45 households with a total of 87 cats randomized to the fipronil-methoprene combination group (40.0% single-cat households). Gender distribution, age ranges, and body weights were generally similar between the groups (Table 1). The youngest cats enrolled in the study were 12 weeks of age in the fluralaner group and 13 weeks of age in the fipronil-methoprene group; 10.7% of fluralaner cats and 11.5% of fipronil-methoprene cats were less than 26 weeks. Several breeds were enrolled, but the most common breeds were domestic shorthair cats (64.7% of fluralaner cats and 75.9% of fipronil-methoprene cats), domestic long hair cats (15.2 and 3.4%), domestic medium hair cats (6.7 and 2.3%), and Siamese pure and crossbreeds (2.7 and 9.2%). Other breeds observed in lower frequencies (less than 5% of either treatment group) included pure and crossbreeds Siamese, Ragdoll, Himalayan, Persian, Manx, Bengal, Maine Coon, and Turkish Angora.

**Table 1:** Demographics of enrolled cats and distribution of numbers of cats in each household

		Fluralaner topical solution (*n* = 224)	Fipronil/(S)-methoprene spot-on solution (*n* = 87)
Age (years)	Mean (SD)	5.0 (4.40)	4.7 (4.30)
	Range	0.2^a^–16.9	0.2^b^–19.4
Weight (lb)	Mean (SD)	9.7 (3.20)	10.2 (3.86)
	Range	2.6–16.9	2.6–25.3
Gender	Female, Intact	27 (12.1%)	7 (8.0%)
	Female, Spayed	93 (41.5%)	35 (40.2%)
	Male, Intact	15 (6.7%)	13 (14.9%)
	Male Neutered	89 (39.7%)	32 (36.8%)
Distribution of household sizes (no. of cats)			
	1	50 (43.1%)	18 (40.0%)
	2	40 (34.5%)	21 (46.7%)
	3	15 (12.9%)	1 (2.2%)
	4	6 (5.2%)	1 (2.2%)
	5	5 (4.3%)	4 (8.9%)

^a^In the fluralaner group, the youngest cats enrolled were 12 weeks of age; 24 (24/224 = 10.7%) were less than 26 weeks of age

^b^In the fipronil-methoprene group, the youngest cats enrolled were 13 weeks of age; 10 (10/87 = 11.5%) were less than 26 weeks of age

In both treatment groups, primary cats occasionally missed visits during this field study, and therefore did not have flea count data generated. In the fluralaner group, there were there were eight primary cats that missed at 8 weeks and nine primary cats that missed at 12 weeks. In the fipronil-methoprene combination group, there were four primary cats that missed at 4 weeks, six primary cats that missed at 8 weeks, and nine primary cats that missed at 12 weeks. In addition, flea count data were collected, but excluded from flea efficacy calculations in both groups for reasons including inaccurate dosing, bathing prior to flea count, insecticidal treatment of the household, and/or administration of other insecticides to the cat. In the fluralaner group, flea count data were excluded from two primary cats at 4 weeks, from two primary cats at 8 weeks, and from two primary cats at 12 weeks. In the fipronil-methoprene combination group, flea count data were excluded from one primary cat at 4 weeks, from one primary cat at 8 weeks, and from two primary cats at 12 weeks.

**Fig. 1:**
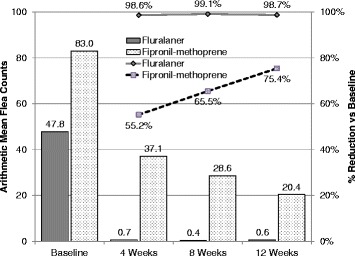
Arithmetic mean flea counts and percent reduction from baseline to weeks 4, 8 and 12 for topical fluralaner or fipronil/(*S*)-methoprene-treated cats (bars indicate arithmetic flea counts; lines indicate percentage reductions from baseline)

In the fluralaner group, reductions in geometric mean counts at 4 weeks (99.1%), 8 weeks (99.5%), and 12 weeks (99.0%) were all statistically significantly different from baseline (all *P* < 0.0001) (Table 2). Arithmetic mean flea count reductions in the fluralaner group were 98.6, 99.1 and 98.7% on weeks 4, 8 and 12, respectively (Table 2, Fig. 1, all *P* < 0.0001). The percentages of individual fluralaner-treated primary cats with a ≥ 90% reduction in flea burden from baseline were 93.9% at 4 weeks, 96.2% at 8 weeks, and 93.3% at 12 weeks. The percentages of individual fluralaner-treated primary cats with 0 fleas detected (i.e. a 100% reduction) were 80.7% at 4 weeks, 88.7% at 8 weeks, and 80.0% at 12 weeks. The maximum 12-week flea count on a primary cat in the fluralaner group was 11 fleas. This was the only one of 105 fluralaner-treated primary cats with a 12-week burden of 10 or more fleas.

**Table 2:** Flea count data for primary cats in the fluralaner topical and fipronil/(*S*)-methoprene groups. No efficacy comparison was performed at V1 and no primary cats had 90% reduction or were flea-free at V1

	Visit 1 (enrollment)	Visit 2 (Week 4, Day 28)	Visit 3 (Week 8, Day 56)	Visit 4 (Week 12, Day 84)
Number of primary cats				
Fluralaner topical solution	116	114	106	105
Fipronil/(S)-methoprene spot-on solution	45	40	38	34
Arithmetic mean flea count (95% CI)				
Fluralaner topical solution	47.8 (36.5–59.1)	0.7 (0.1–1.3)	0.4 (0.0–1.0)	0.6 (0.3–0.9)
Fipronil/(S)-methoprene spot-on solution	83.0 (24.7–141.2)	37.1 (14.5–59.7)	28.6 (14.3–42.9)	20.4 (10.0–30.8)
*P*-value for comparison^a^	*t*_(158.9)_ = -1.71, *P* = 0.0899	*t*_(160.5)_ = -5.17, *P* < 0.0001	*t*_(155.8)_ = -6.35, *P* < 0.0001	*t*_(148.4)_ = -6.88, *P* < 0.0001
% efficacy (reduction from baseline) based on arithmetic means				
Fluralaner topical solution	na	98.6	99.1	98.7
*P-*value for comparison to baseline^a^	na	*t*_(158.5)_ = 5.51, *P* < 0.0001	*t*_(159.2)_ = 4.74, *P* < 0.0001	*t*_(159.1)_ = 4.50, *P* < 0.0001
Fipronil/(S)-methoprene spot-on solution	na	55.2	65.5	75.4
*P*-value for comparison to baseline^a^	na	*t*_(160.4)_ = 3.49, *P* = 0.0006	*t*_(159.9)_ = 3.50, *P* = 0.0006	*t*_(159.6)_ = 3.75, *P* = 0.0002
Geometric mean flea count (95% CI)				
Fluralaner topical solution	28.0 (23.2–33.8)	0.2 (0.1–0.4)	0.1 (0.0–0.2)	0.3 (0.2–0.4)
Fipronil/(S)-methoprene spot-on solution	28.0 (19.2–40.9)	15.0 (9.5–23.3)	9.4 (5.2–16.4)	6.8 (3.6–12.1)
*P*-value for comparison^a^	*t*_(139.9)_ = 0.07, *P* = 0.9474	*t*_(139.7)_ = -17.56, *P* < 0.0001	*t*_(144.7)_ = -13.23, *P* < 0.0001	*t*_(139.3)_ = -10.80, *P* < 0.0001
% efficacy (reduction from baseline) based on geometric means				
Fluralaner topical solution	na	99.1	99.5	99.0
*P*-value for comparison to baseline^a^	na	*t*_(151.9)_ = 32.41, *P* < 0.0001	*t*_(150.8)_ = 30.60, *P* < 0.0001	*t*_(147.1)_ = 29.81, *P* < 0.0001
Fipronil/(S)-methoprene spot-on solution	na	46.5	66.6	75.8
*P*-value for comparison to baseline^a^	na	*t*_(159.3)_ = 3.96, *P* = 0.0001	*t*_(155.9)_ = 6.11, *P* < 0.0001	*t*_(158.4)_ = 7.68, *P* < 0.0001
% of primary cats with at least 90% reduction from baseline flea count				
Fluralaner topical solution	na	93.9	96.2	93.3
Fipronil/(S)-methoprene spot-on solution	na	10.0	31.6	38.2
*P*-value for comparison^b^	na	*Z* = -10.17, *P* < 0.0001	*Z* = -7.26, *P* < 0.0001	*Z* = -6.96, *P* < 0.0001
% of flea-free primary cats				
Fluralaner topical solution	na	80.7	88.7	80.0
Fipronil/(S)-methoprene spot-on solution	na	5.0	21.1	23.5
*P*-value for comparison^b^	na	*Z* = -8.45, *P* < 0.0001	*Z* = -7.87, *P* < 0.0001	*Z* = -6.05, *P* < 0.0001

*Abbreviation*: *na* value or calculation is not applicable

^a^ *P*-value for comparison of model least squares means parameter estimates

^b^ *P*-value for comparison of percentages using non-parametric asymptotic approach and Standardized Statistic

At each post-treatment assessment of the fipronil-methoprene combination group, geometric mean flea count reductions from baseline were significant (all *P* ≤ 0.0001), but at no point did they reach 90% (46.5% at 4 weeks, 66.6% at 8 weeks, and 75.8% at 12 weeks). Similarly, arithmetic mean flea count reductions from baseline were significant (*P* ≤ 0.0006), but at no point did they reach 90% (55.2% at 4 weeks, 65.5% at 8 weeks, and 75.4% at 12 weeks). The percentages of individual fipronil-methoprene-treated primary cats with a ≥ 90% reduction in flea burden from baseline were 10.0% at 4 weeks, 31.6% at 8 weeks, and 38.2% at 12 weeks. The percentages of individual fipronil-methoprene-treated primary cats with 0 fleas detected (i.e. a 100% reduction) were 5.0% at 4 weeks, 21.1% at 8 weeks, and 23.5% at 12 weeks. In six fipronil-methoprene combination product-treated households, the counts at 12 weeks were higher than at enrollment, and at least one clinic in each of Alabama, Illinois, Louisiana, Maine, and Missouri identified a primary fipronil-methoprene-treated cat with at least 20 fleas-single cat households represented 70% of the primary cats with at least 20 fleas at 12 weeks.

In both groups, there was improvement in all signs of flea allergy dermatitis (Table 3). The most common manifestation of FAD observed in study cats was alopecia, which at enrollment was recorded for 60 of 224 (26.8%) cats in the fluralaner group and 18 of 84 (21.4%) cats in the fipronil-methoprene combination group. At 12 weeks, the percentage of eligible cats in which this sign was seen to have resolved was 84.4% in the fluralaner and 50.0% in the fipronil-methoprene combination group.

**Table 3:** Summary of alleviation in each sign of flea allergy dermatitis in cats

Sign		Fluralaner topical solution	Fipronil/(*S*)-methoprene spot-on solution
Erythema	Number of cats with sign at initial exam that were also eligible for re-examination at 12 weeks	26	6
	% of cats with lesion resolved at 12-week re-examination	80.8	33.3
Alopecia	Number of cats with sign at initial exam that were also eligible for re-examination at 12 weeks	45	12
	% of cats with lesion resolved at 12-week re-examination	84.4	50.0
Papules	Number of cats with sign at initial exam that were also eligible for re-examination at 12 weeks	6	1
	% of cats with lesion resolved at 12-week re-examination	100.0	100.0
Scales	Number of cats with sign at initial exam that were also eligible for re-examination at 12 weeks	15	2
	% of cats with lesion resolved at 12-week re-examination	100.0	100.0
Crusts	Number of cats with sign at initial exam that were also eligible for re-examination at 12 weeks	24	7
	% of cats with lesion resolved at 12-week re-examination	95.8	57.1
Excoriation	Number of cats with sign at initial exam that were also eligible for re-examination at 12 weeks	25	4
	% of cats with lesion resolved at 12-week re–examination	100.0	75.0

Across the 12 weeks of the study in all cats of both treatment groups and for an additional 3 weeks following the second treatment of fluralaner-treated cats, there were no serious adverse events. All adverse events were unremarkable throughout the study. A review of study records and owner diaries showed the most common adverse event in each group to be vomiting, affecting 7.6% of fluralaner-treated cats and 6.9% of fipronil-methoprene-treated cats. Other adverse events occurred at generally similar rates in both groups, although pruritus was reported in 11.5% of fipronil-methoprene-treated cats and 5.4% of fluralaner-treated cats; diarrhea and decreased appetite were reported in more fluralaner-treated cats (4.9 and 3.6%, respectively) than fipronil-methoprene-treated cats (1.1 and 0.0%, respectively) (Table 4).

**Table 4:** Percent of cats experiencing most common adverse events reported during the study

	Fluralaner topical solution (*n* = 224 cats)	Fipronil/(*S*)-methoprene spot-on solution (*n* = 87)
Vomiting	7.6	6.9%
Pruritus	5.4	11.5%
Diarrhea	4.9	1.1%
Alopecia	4.9	4.6%
Decreased appetite	3.6	0.0%
Lethargy	3.1	2.3%
Scabs/Ulcerated lesions	2.2	3.4%

Both groups underwent a detailed blood count and blood and urine analysis and the results were unremarkable, with no evidence suggesting a pathologic trend, and only occasional isolated departures from normal reference ranges in blood and urine analyses. There were no clinically relevant differences between the results of the two groups.

## Discussion

This 12-week field study found that a single topical administration of fluralaner provided a greater than 99% (by geometric mean; ≥ 98.6% by arithmetic mean) flea control efficacy over 12 weeks. Under equivalent field challenge conditions, flea control efficacy never exceeded 90% in the positive control group treated three times with a fipronil/(*S*)-methoprene combination. The results of this field study in cats align with the results of three similarly designed studies in dogs - two investigating the chewable fluralaner formulation, and one investigating the use of the same topical formulation used in this study [10, 11, 13]. In all of the dog fluralaner field studies, a single treatment/application has resulted in a > 99% reduction in geometric mean live flea counts within one month, and sustained flea count reductions of > 99% through 12 weeks post-treatment.

Both fluralaner and fipronil-methoprene combination products were well tolerated in this study. There were no detectable effects of either product on clinical pathology tests (baseline and week 12 for fipronil-methoprene cats; baseline, week 12, and week 15 for fluralaner cats).

There are few published reports of clinical field studies that focus on flea control measures in cats, with only one in which enrollments were from cat-only (i.e. no dogs) households [16]. In that study, cats received two sequential monthly treatments with orally administered spinosad or topically applied selamectin. At the final assessment 30 days after the second treatment (Day 60), arithmetic mean flea count reductions from pre-treatment levels were 98.7 and 98.2% in the spinosad and selamectin groups, respectively, with 91.0 and 87.9% of cats in each group free of fleas. Those efficacy findings align with observations for fluralaner single-treatment efficacy at 8 weeks in this study, although the fipronil-methoprene group efficacy results reported here appear to lag behind other treatments.

The flea control efficacy observed in this study following treatment with a fipronil-methoprene combination product was inconsistent. Six primary cats treated with fipronil-methoprene combination had flea counts that were higher at 12 weeks than at enrollment; five owners withdrew from the study prior to 12 weeks because of perceived inefficacy; and, 23.5% of primary cats were flea free while 38.2% had a 90% or greater reduction in flea count at week 12. These observations are consistent with previous reports describing inadequate fipronil flea control efficacy [13, 17, 18], and to the potential need for further investigation into explanations for this weaker than expected performance.

FAD clinical signs resolved in most fluralaner-treated cats, an observation that is consistent with results following fluralaner treatment of FAD affected dogs. In this field study the diagnosis of FAD was based exclusively on clinical signs and it is possible that some of the clinical signs observed at enrollment were not caused by flea bites. Studies using more detailed diagnostic methods in dogs have observed a greater response following effective flea treatment [12, 19].

## Conclusions

In conclusion, a single topical administration of fluralaner to cats is well tolerated and highly effective in controlling flea infestations on cats for 12 weeks following administration under typical household conditions.

## Abbreviation

FAD: Flea allergy dermatitis
